# Extreme Early Image Recognition Using Event-Based Vision

**DOI:** 10.3390/s23136195

**Published:** 2023-07-06

**Authors:** Abubakar Abubakar, AlKhzami AlHarami, Yin Yang, Amine Bermak

**Affiliations:** Division of Information and Computing Technology, College of Science and Engineering, Hamad Bin Khalifa University, Doha P.O. Box 34110, Qatar; alkuzama.alharmi@hotmail.com (A.A.); yyang@hbku.edu.qa (Y.Y.); abermak@hbku.edu.qa (A.B.)

**Keywords:** convolutional neural network, early image recognition, event-based camera, sensors

## Abstract

While deep learning algorithms have advanced to a great extent, they are all designed for frame-based imagers that capture images at a high frame rate, which leads to a high storage requirement, heavy computations, and very high power consumption. Unlike frame-based imagers, event-based imagers output asynchronous pixel events without the need for global exposure time, therefore lowering both power consumption and latency. In this paper, we propose an innovative image recognition technique that operates on image events rather than frame-based data, paving the way for a new paradigm of recognizing objects prior to image acquisition. To the best of our knowledge, this is the first time such a concept is introduced featuring not only extreme early image recognition but also reduced computational overhead, storage requirement, and power consumption. Our collected event-based dataset using CeleX imager and five public event-based datasets are used to prove this concept, and the testing metrics reflect how early the neural network (NN) detects an image before the full-frame image is captured. It is demonstrated that, on average for all the datasets, the proposed technique recognizes an image 38.7 ms before the first perfect event and 603.4 ms before the last event is received, which is a reduction of 34% and 69% of the time needed, respectively. Further, less processing is required as the image is recognized 9460 events earlier, which is 37% less than waiting for the first perfectly recognized image. An enhanced NN method is also introduced to reduce this time.

## 1. Introduction

The rapid development and integration of artificial intelligence with image sensors has revolutionized machine vision. Real-time image recognition is an essential task for many applications, such as emerging self-driving vehicles. These applications require continuous and fast image acquisition combined with computationally intensive machine learning techniques such as image recognition. The use of frame-based cameras in such applications introduces four main challenges including: (1) high bandwidth consumption due to large amounts of data transmission; (2) large memory requirement for data storage prior to processing; (3) computationally expensive algorithms for real-time processing; (4) large power and energy consumption for continuous data transmission and processing.

Unlike frame-based cameras, which are based on the concept of sequentially acquiring frames, an event-based imager generates a series of asynchronous events reflecting the change in light intensity per pixel. This concept is derived from the operation of biological vision systems; specifically, the retina. The first functional model to simulate the magno cells of the retina was introduced in 2008 by Tobi’s group under the term dynamic vision sensors (DVSs) [1]. An event-based imager, also known as a silicon retina, dynamic vision sensor (DVS), or neuromorphic camera, is a biologically inspired vision system that acquires visual information in a different way than conventional cameras. Instead of capturing absolute brightness of full images at a fixed rate, these imagers asynchronously respond to changes in brightness per pixel. An “event” is generated if the change in brightness at any pixel surpasses a user-defined threshold. The output of the sensor is a series of digital events <(*x*, *y*), *I*, *t*> (or spikes) that includes the pixel’s address (x,y), time of event (*t*), and sign of change in brightness (*I*) [2,3,4,5,6]. Event-based imagers present several major advantages over normal cameras, including lower latency and power consumption, as well as higher temporal resolution and dynamic range [6]. This allows them to record well in both very dark and very bright scenes.

The novel design of event-based imagers introduces a paradigm shift in the camera sensor design. However, because these sensors produce different data outputs, current image recognition algorithms for conventional cameras are not suitable for them. To our knowledge, there does not yet exist an ideal solution for extracting information from the events produced by the sensors. A few image recognition algorithms have been introduced in the literature, but they are still far from mature [7].

Event-based imagers are capable of overcoming the lost time between frames; hence, they are able to process the information in the “blind” time between each frame. The data collected from event-based imagers have a significantly different structure compared to frame-based imagers. To effectively extract useful information and utilize the full potential of the asynchronous, sparse, and timed data collected from event-based images, we need to either design new processing algorithms or adapt and/or re-design existing vision algorithms for this purpose. The first approach is expected to provide more reliable and accurate results; yet, most existing solutions in the literature follow the second approach. In particular, events are either processed as: (i) a single event, which updates the output of the algorithm based on every new event received, minimizing latency, or (ii) a group of events, which updates the output of the algorithm after a group of events has arrived by using a sliding window. These methodologies are selected based on how much additional information is required to perform a task. In some cases, a single event cannot be useful on its own due to having little information or a lot of noise; hence, additional information is required in the form of either past events or new knowledge.

Frame-based algorithms have advanced to learn features from data using deep learning. For instance, convolutional neural networks (CNNs) are a mature approach for object detection; therefore, many works utilize CNNs and apply them to event-based data. Such works can be divided into two categories: methods that use a frame-based CNN directly [8,9,10,11] and methods that rewire a CNN to take advantage of the structure of event-based data [11,12]. Recognition can sometimes be applied to events that are transformed to frames during inference [13,14], or by converting a trained neural network to a spiking neural network (SNN) which can operate on the event-based data directly [15,16,17].

In this paper, we propose an innovative image recognition technique that operates on image events rather than frame-based data, paving the way to a new paradigm of recognizing objects prior to image acquisition. A faster object recognition technique is proposed using event-based imagers instead of frame-based imagers to achieve on-the-fly object recognition. Using event sequences from the event-based imager, objects are recognized on-the-fly before waiting for the full-frame image to appear. To the best of our knowledge, this is the very first time such a concept is being introduced, other than our initial work in [18], which not only achieves an early image recognition, but also addresses all four challenges mentioned previously, as less data will be transmitted and processed, enabling faster object recognition for applications with real-time constraints. This work explores dataset acquisition and labeling requirements and methodologies using an event-based imager (Celepixel). It adapts existing frame-based algorithms to implement early image recognition by testing the concept on both publicly available datasets and the datasets collected in this work using Celepixel. It also explores enhancing the algorithms to achieve even earlier image recognition.

The rest of the paper is organized as follows. Section 2 introduces the concept of early recognition. Section 3 explains and analyzes the datasets used to validate our concept. Section 4 describes the proposed early recognition algorithm and testing metrics. Section 5 presents and analyzes our experimental results. Section 6 describes an enhanced early recognition method and presents the results. Finally, Section 7 concludes this work.

## 2. Early Recognition

**Figure 1:**
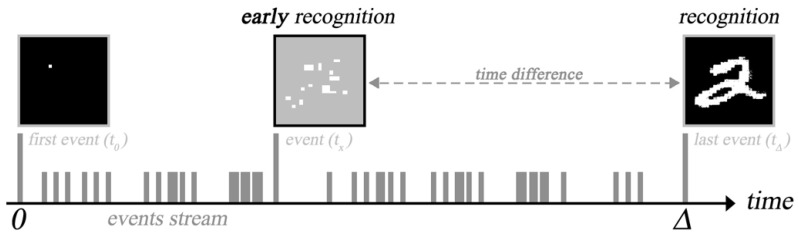
Concept of early image recognition.

The main idea of this work is to utilize event-based imagers to achieve early image recognition, as illustrated in Figure 1. The concept of early recognition is defined as accurate image recognition before the last event is received from the event-based imager. In other words, this implies the ability to detect an object without waiting for the full picture to be captured. The idea is derived from the main feature of event-based imagers, where each pixel fires asynchronously in response to any brightness change. An “event” is generated if the change in brightness at any pixel surpasses a user-defined threshold. The output of the sensor is a series of digital events in the form of 〈(x,y),I,t〉 that includes the pixel’s location (x,y), time of event (*t*), and the sign of change in brightness (*I*). We aim to process these events as they arrive in real time to perform recognition, which enables us to process the data faster, as there is no longer frame rate limitations; meanwhile, redundant background data are also eliminated.

To achieve early recognition, existing frame-based algorithms can be used and adapted to work with event data. The data used to train the algorithm can be normal images (frame-based) or event data. The data need to be pre-processed to match the input of the algorithm selected, which includes operations such as resizing, compression, noise reduction, etc.

To test the concept, the events are fed to the algorithm as they arrive, and two main metrics are evaluated:

- First Zero Event (FZE): the first time that the algorithm is able to obtain a zero error rate for the input (this condition starts with the first pixel change and ends before the full image is detected by the sensor).
- First Perfect Event (FPE): the first time that the algorithm is able to obtain a zero error rate and a confidence level of more than 0.95 (this condition starts after the FZE is detected).

The first zero metric is used to determine the time when the algorithm can guess what the displayed image will be.

In this work, our collected dataset using CeleX (CeleX-MNIST) in Section 3.1 and five different public datasets (MNIST-DVS [19], FLASH-MNIST [19], N-MNIST [20], CIFAR-10 [21], and N-Caltech-101 [20]), collected using different image sensors, are utilized to perform experiments. Two different types of neural networks (InceptionResNetV2 and ResNet50) are trained on the original images (MNIST [22], CIFAR-10 [23], and Caltech-101 [24]), and then tested on the above-mentioned event-based images to demonstrate the ability of early recognition on event-based data. The recognition is then enhanced by training the same neural network on noisy images, referred to in this work as partial pictures (PP).

## 3. Data Acquisition and Analysis

This section discusses the method to collect each dataset. Moreover, each dataset has different statistical properties that change based on the recording method, sensor sensitivity, and the data being captured. In this section, we explain how the five datasets selected are analyzed to identify their properties and statistical differences, which are summarized in Table 1.

Basic statistical analysis has been performed to identify how long each recording is (in ms) and obtain the total average for each dataset. This helps in calculating how long each saccade (in ms) takes as part of the recording, whether created by either sensor or image movement. Further analysis is conducted to calculate the average number of events per recording, which is affected by the image size, details within each image, and the sensor’s sensitivity. The ON and OFF events average is also calculated. The ranges of the *x*- and *y*-addresses are also calculated to make sure that there are no fault data outside of the sensor size.

The datasets are arranged from least to most complex. MNIST is considered one of the basic datasets as it only includes numbers. CIFAR10 has more details within each image, and yet only contains 10 classes. Caltech101 is the most complex as it contains a large number of classes and each image has detailed objects and backgrounds.

### 3.1. CeleX-MNIST

MNIST-CeleX is collected in this work using the CeleX sensor, which is a 1 Mega Pixel (800 Rows × 1280 Columns) event-based sensor designed for machine vision [25]. It can produce three outputs in parallel: motion detection, logarithmic picture, and full-frame optical flow. The output of the sensor can be either a series of events that are produced in an asynchronous manner or synchronous full-frame images. The sensor can be configured in many modes including: event off-pixel timestamp, event in-pixel timestamp, event intensity, full picture, optical flow, or multi-read optical-flow. Moreover, within the modes, the sensor is able to generate different event image frames: event binary pic, event gray pic, event accumulated gray pic, or event count pic. The output data contain different information depending on the mode selected, including: address, off-pixel timestamp, in-pixel timestamp, intensity, polarity, etc.  [26].

**Figure 2:**
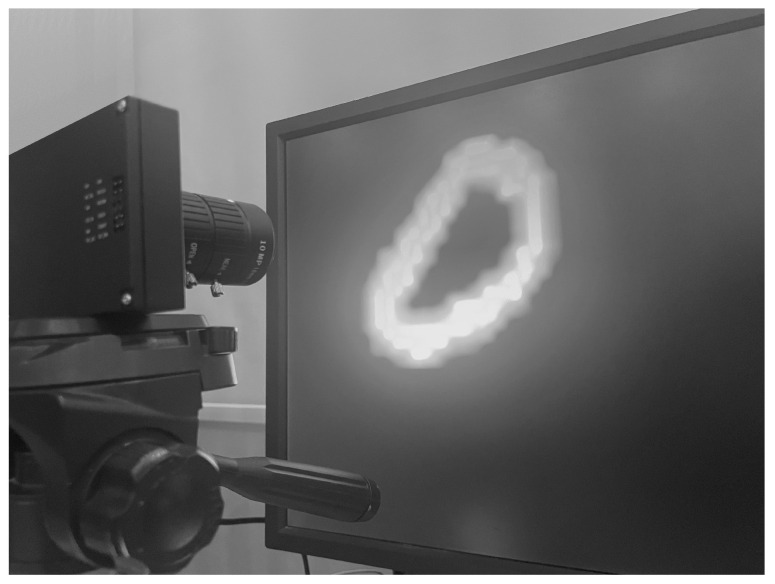
CelePixel data acquisition setup.

**Figure 3:**
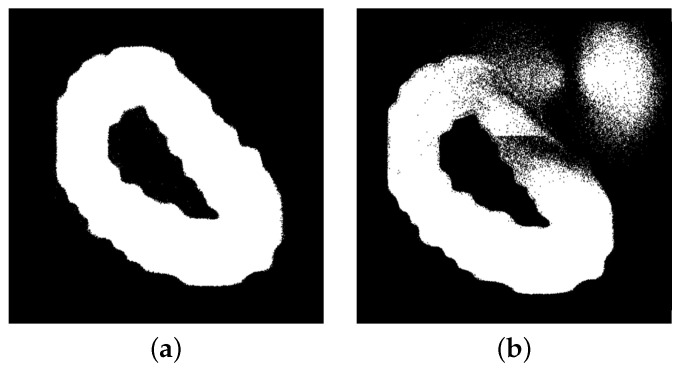
CelePixel data acquisition conditions: (**a**) regular conditions; (**b**) with flickering light.

To collect the dataset, the CeleX sensor is mounted on a base opposite a computer screen that displays the dataset as shown in Figure 2. While collecting the dataset, the environment around the imager must be controlled in order not to allow any glare or reflection from the screen. In Figure 3, the difference between a controlled well-lit environment vs. an environment with flickering lights is displayed. In order to avoid any artifacts and false pixel changes, the same stable conditions should be used throughout the data collection.

To capture the MNIST, the 600 training samples of MNIST were scaled to fit the sensor sizes and flashed on an LCD screen. As noted in Table 1, both the total time average and Saccade time average of the recordings was 631 ms, as we only flashed the image once. The size of the full sensor is shown in the min and max values; however, the image is only shown on an estimate of 800 × 800 of the sensor.

Algorithm 1 explains in detail the methodology followed in this work for data acquisition. Once the mode is selected, each image is scaled to 800 × 800, then flashed once and followed by a black image. Resetting the scene to black allows the sensor to detect the change in the flashed image only. The dataset consists of 600 recordings for 10 classes (digits 0–9). Each collected event in the recording includes five pixel information:

- Row address: range 0 to 799;
- Column address: range 0 to 1279;
- Event timestamp: range 0 to 2${}^{31}$ in microseconds;
- Intensity polarity: −1: OFF, 0: unchanged, +1: ON;
- Intensity: range 0 to 4095.

### 3.2. MNIST-DVS

The dataset [19] was created using a 128 × 128 event-based imager [27]. To obtain the dataset, the original test set (10,000 images) of the MNIST dataset was scaled to three different sizes (4, 8, and 16). Each scaled digit was then displayed slowly on an LCD screen and captured by the imager.

The dataset consists of 30,000 recordings, 10,000 per scale, for 10 classes (digit 0–9). Each collected event in the recording includes four pixel attributes:

- Row address: range 0 to 127;
- Column address: range 0 to 127;
- Event timestamp: in microseconds;
- Intensity polarity: −1: OFF, +1: ON.

**Algorithm 1** CeleX-MNIST dataset acquisition using CelePixel
**Sensor Mode:** 
intensity**Picture Mode:** 
gray picture**Input:** 
image matrix**Output:** 
raw events     *INITIALIZATION*:  1:load dataset     
*LOOP PROCESS*
  2:**for** i=0 to 599 **do**  3:   display black image  4:   pause for 0.2 s  5:   start collecting events  6:   scale image to 800 × 800  7:   display image  8:   label event  9:   pause for 0.2 s10:   stop collecting events11:   display black image12:   pause for 0.2 s13:   export collected events


As explained in [19], to capture the MNIST-DVS, the 10,000 training samples of MNIST were scaled to three different sizes and displayed slowly on an LCD screen. It can be observed from Table 1 that as the scale of the image increases, the number of events produced by the imager increase as well, ranging from 17,011 events per image to 103,133 events. However, the recording period is almost similar for all three scales with an average of 2347 ms. As the number of saccade movements performed to create the movement was not mentioned in [19], we assumed that one recording is one saccade.

### 3.3. FLASH-MNIST

The dataset [19] is created using a 128 × 128 event-based imager [27]. To obtain the dataset, each of the 70,000 images in the original MNIST dataset is flashed on a screen. In particular, each digit is flashed five times on an LCD screen and captured by the imager.

The dataset consists of 60,000 training recordings and 10,000 testing recordings, with 10 classes (digits 0–9). Each collected event in the recording includes four pieces of pixel information:

- Row address: range 1 to 128;
- Column address: range 1 to 128;
- Event timestamp: in microseconds;
- Intensity polarity: 0: OFF, +1: ON.

The Flash-MNIST dataset is divided into training and testing recordings. The average total time for both datasets is 2125 ms. As explained in [19], each image is flashed for five times on the screen. Each saccade duration is an average of 425 ms.

### 3.4. N-MNIST

**Figure 4:**
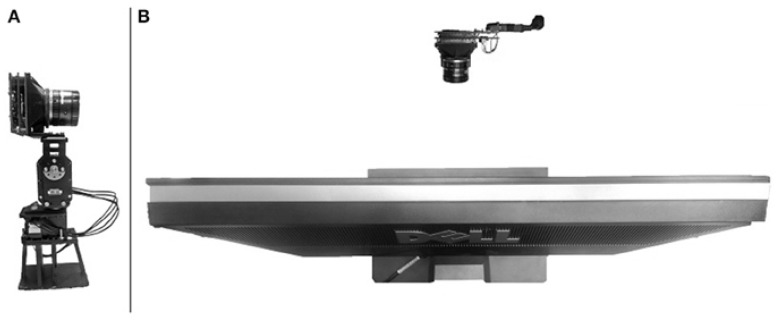
(**A**) ATIS mounted on a motorized pan-tilt unit. (**B**) ATIS positioned in front of an LCD monitor [20].

The dataset [20] is created using a 240 × 180 event-based imager ATIS [28]. To capture the dataset, this work uses an imager mounted on a motorized pan-tilt unit. The imager is mounted on the motorized system consisting of two motors and positioned in front of an LCD monitor, as shown in Figure 4. To create movements, the imager moves up and down (three micro-saccades), creating motion and capturing the images on the monitor.

The original images were resized, while maintaining the aspect ratio, to ensure that the size does not exceed 240 × 180 pixels (ATIS size), before being displayed on the screen. The MNIST were resized to fill up 28 × 28 pixels on the ATIS sensor. The dataset consists of 60,000 training recordings and 10,000 testing recordings for 10 classes (digits 0–9). Each collected event in the recording includes four aspects of pixel information:

- Row address: range 1 to 34;
- Column address: range 1 to 34;
- Event timestamp: in microseconds;
- Intensity polarity: +1: OFF, +2: ON.

The imager used to record N-MNIST [20] is 240 × 180; however, the dataset is recorded only with 34 × 34 pixels. The dataset is divided into training and testing recordings. Each recording contains three saccades, each with a duration of 102 ms, leading to a total recording for a single image of 306 ms. Compared to MNIST-DVS and FLASH-MNIST, this dataset has a very low average number of events considering its small pixel size.

### 3.5. CIFAR-10

**Figure 5:**
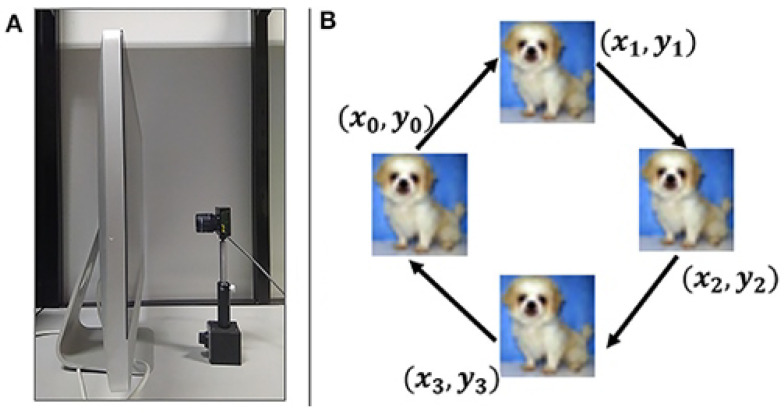
(**A**) Recording setup of sensor positioned in front of an LCD monitor. (**B**) Image movement sequence on screen [21].

The dataset [21] is created using a 128 × 128 event-based imager [1], as shown in Figure 5A. To capture the dataset, a repeated closed-loop smooth (RCLS) image movement method is used. The recording setup is placed inside a dark compartment and does not require any motors or control circuits. The recording starts with an initialization stage that loads all the data. Then, each loop in the RCLS has four moving paths at an angle of 45 degrees, as shown in Figure 5B, and the full loop is repeated six times. A 2000 ms wait is required between every image so that the next recording is not affected.

The original images were upsampled, while maintaining the aspect ratio, from 32 × 32 to 512 × 512. The dataset consists of 10,000 recordings, as images were randomly selected from the original dataset with 1000 image per class. Each collected event in the recording includes four pixels of information:

- Row address: range 0 to 127;
- Column address: range 0 to 127;
- Event timestamp: in microseconds;
- Intensity polarity: −1: OFF, +1: ON.

In this dataset [21], the recordings are created by moving the image on the screen to four locations and repeating this loop six times; hence, having 24 saccades. Each saccade lasts for 54 ms and adds up to an average total time of 1300 ms per recording. The event count is very high compared to MNIST-DVS which has the same 128 × 128 size. The reason behind the increase in events generated by the imager is that the CIFAR10 dataset has images with complex details and backgrounds, unlike MNIST which only has numbers.

### 3.6. N-Caltech 101

The dataset [20] was created using a 240 × 180 event-based imager ATIS [28]. The dataset was captured using the same recording technique explained for N-MINST.

The original images vary in size and aspect ratio. However, every image was scaled as large as possible, while maintaining the aspect ratio, to ensure the size did not exceed 240 × 180 pixels (ATIS size) before being displayed on the screen. The dataset consists of 8709 recordings for 10 classes. Each collected event in the recording includes four pixels of information:

- Row address: range 1 to 180;
- Column address: range 1 to 240;
- Event timestamp: in microseconds;
- Intensity polarity: +1: OFF, +2: ON.

This dataset was recorded using the same method and imager as the N-MNIST. However, for N-Caltech 101 [20], the full imager size is used, as these images are bigger and have more details. Each recording contains three saccades, each with a duration of 100 ms, which creates a total recording for a single image of 300 ms. Due to using the full sensor size and the number of details in these images, it is noticed that the number of events is almost 28 times more than N-MNIST.

## 4. Early Recognition Method

An existing image recognition algorithm was used with a group events methodology, as described in Section 1, which waits for a group of events to occur then passes the data to the recognition algorithm. This section describes the network architectures used for early image recognition as well as the testing methodology and metrics.

### 4.1. Neural Network

Two network architectures are used to process the data.

#### 4.1.1. InceptionResNetV2

**Figure 6:**
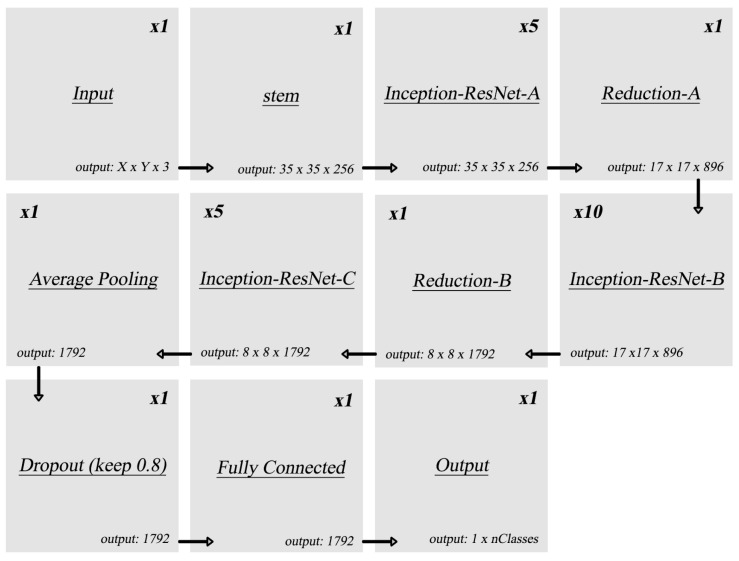
Original InceptionResNetV2 architecture.

The network architecture used is shown in Figure 6 [29], which consists of stem block, Inception-Resnet (A, B, and C) with reduction, average pooling, dropouts, and fully connected output layers. The InceptionResNetV2 is pre-trained on ImageNet [30], which consists of 1.2 million images. The original network has an output of 1000 classes; hence, a new output layer is added and trained on the original dataset (MNIST or CIFAR-10) to be tested. All inputs are pre-processed by being resized to match the image size that the network has been trained on.

#### 4.1.2. ResNet50

**Figure 7:**
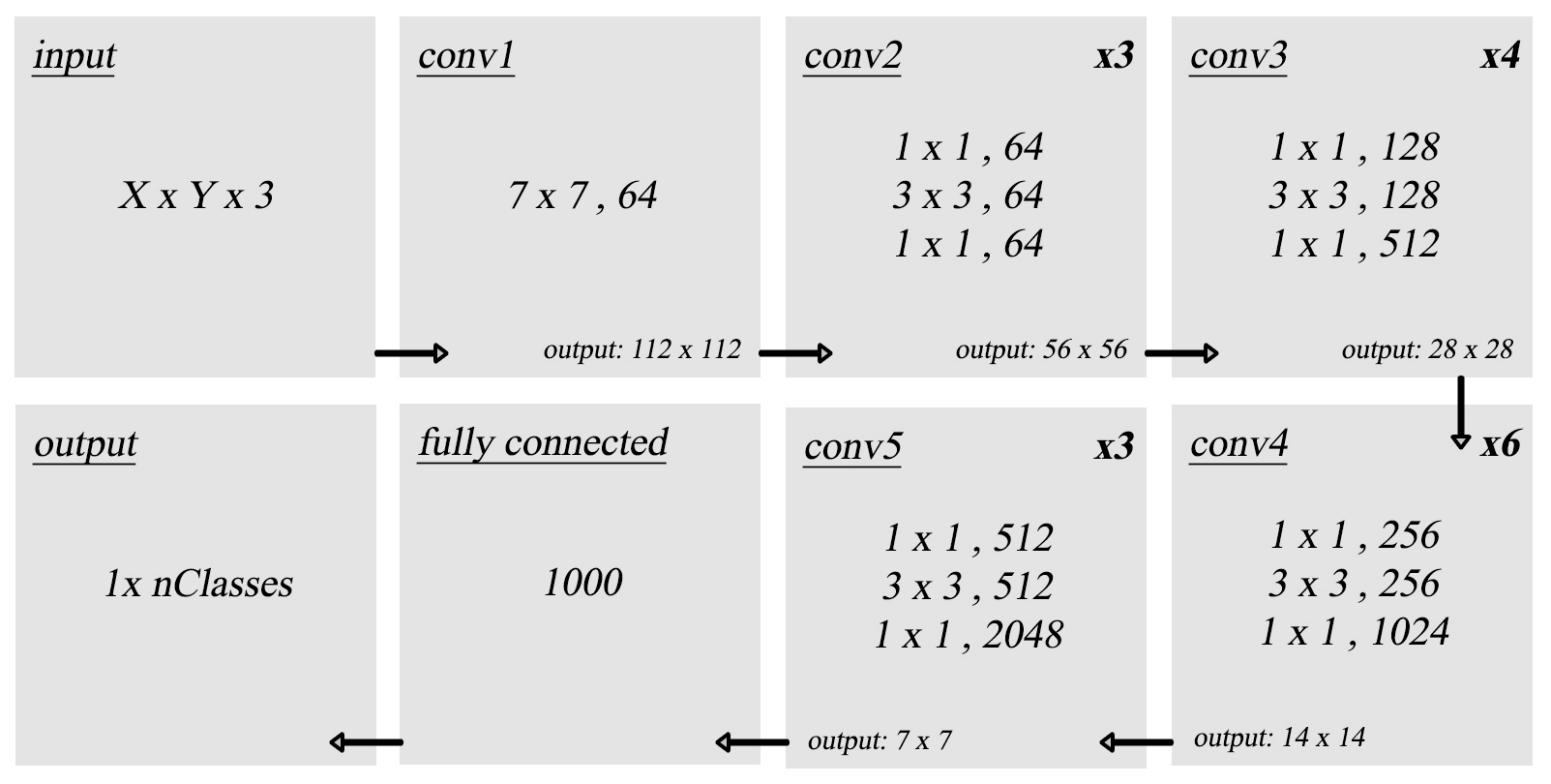
Original ResNet50 architecture.

The network architecture used is shown in Figure 7 [31], which consists of convolutional, average pooling, and fully connected output layers. The ResNet50 is pre-trained on ImageNet [30], which consists of 1.2 million images. The weights of the ResNet50 convolutional layers are frozen, while the fully connected output layer is removed. A new output layer is added and trained on the original dataset (Caltech-101) to be tested. All inputs are pre-processed by being resized to match the image size that the network has been trained on.

### 4.2. Testing on Collected Events

- Input Preprocessing
  The size of the events collected is not fixed, and thus it is resized to match the input of the neural network architecture. As the dataset is very big, the recognition is conducted after every group of event, noting that time will be more accurate if event-by-event methodology was used instead of group events.
- Testing and Metrics
  Algorithm 2 describes the testing algorithm. The events are feedforwarded to the neural network model after each group of events and then two main metrics, FZE and FPE, explained in Section 2, are calculated.

**Algorithm 2** Event-based dataset testing on NN architecture
**Input:** 
raw events from dataset**Output:** 
FZE, FPE     *INITIALIZATION*:  1:load trained weight of network model  2:load raw events  3:initialize a matrix with zeros (image)     *DATA PREPROCESSING*:  4:resize raw events to match input     
*LOOP PROCESS*
  5:**for** i=1 to events count **do**  6:     update the image with event *i*  7:     **if** (i mod #eventPerGroup) = 0 **then**  8:       test the image in the neural network  9:       **if** class_code is correct & FZE flag = 0 **then**10:          FZE equals *i*11:          FZE flag equals 112:       **if** class_code is correct & probability >= 0.95 & FPE flag = 0 **then**13:          FPE equals *i*14:          FPE flag equals 115:calculate the difference between FZE and FPE


## 5. Early Recognition Analysis and Results

### 5.1. CeleX-MNIST

As stated in Section 4, the InceptionResNetV2 is trained on the original MNSIT dataset. To calculate its accuracy, the trained model is tested on 10,000 images of the original MNIST dataset and reports an accuracy of 99.07%.

The 600 raw event-based recording images are selected from the CeleX-MNIST dataset. The set contains recordings with events collected on an average time of 631 (ms) and an average event count of 420,546 per recording. The size of the events collected is 800 × 1280 pixels, so it is first cropped to 800 × 800 then resized to 28 × 28 to match the input of the InceptionResNetV2 architecture. As the dataset is very big, the recognition is conducted every 1000 events. The results are analyzed as per the testing metrics described in Table 2.

**Figure 8:**
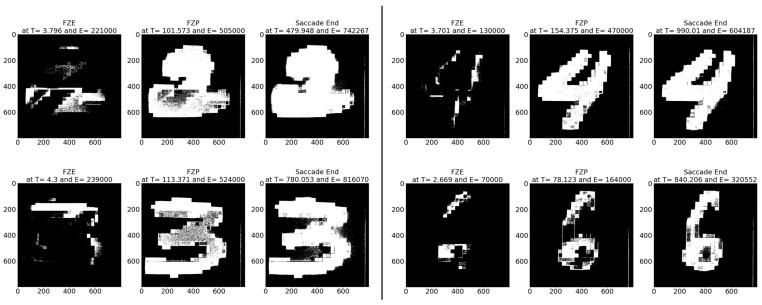
Sample CeleX-MNIST test images at event (**left**) FZE, (**middle**) FPE, (**right**) saccade end.

**Figure 9:**
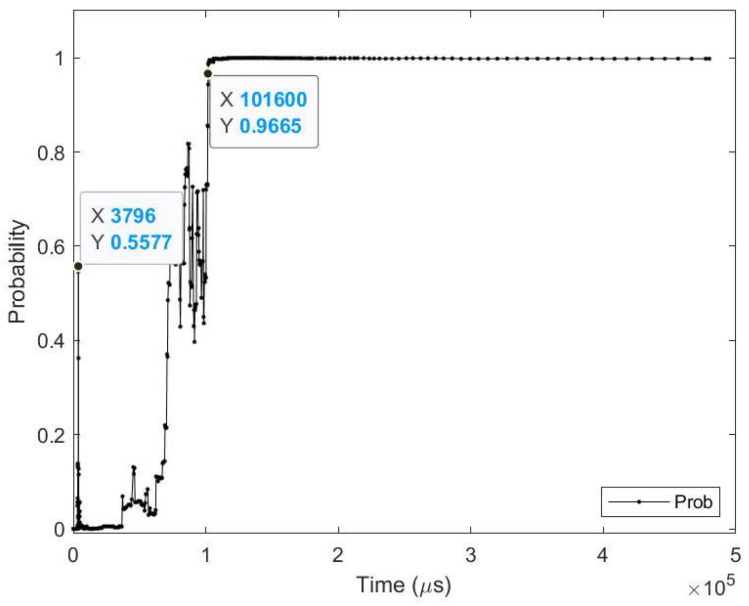
Testing result of CeleX-MNIST class 2 image using time sequence.

- **Average Results:** As shown in Table 3, on average the images are detected 14.81 (ms) earlier, which is around 28.78% before the full image is displayed. In terms of event sequence, the image is detected around 18.92% before, or 32,558 events before the full image is accumulated.
  Table 4 summarizes the testing metrics per image category; only three categories are reported here for reference.

- **Sample test image results:** Figure 8 illustrates sample test images at the first zero, first perfect, and saccade events. It can be noticed that at the FZE images, the details of the image are not yet displayed; however, the network can still recognize the image with an average probability of 56.20%. The probability keeps increasing as more events are processed, as illustrated in Figure 9.
  The result of processing a single raw image file from class (2), which is shown in Figure 8 (first row), is discussed here in detail. The selected raw file contains an event count of 742,268 events which are collected at a duration of 479.95 (ms). As the events are processed, the image is updated and feedforwarded to the InceptionResNetV2 trained network. The network predicts the category of the image and provides probability against the 10 classes. Figure 9 illustrates the probability of the image (black line) against the time sequence. As discussed above, as more events are processed, the probability increases. For this image, the network is able to detect the zero and perfect event, as described in Table 5.

### 5.2. MNIST-DVS

The same trained neural network in Section 5.2 is used to test this datatset.

The 10,000 (scale 16) raw event-based recording images are selected from the MNIST-DVS dataset. The set contains recordings with events collected on an average time of 2411.81 (ms) and an average event count of 103,144 per recording. The size of the events collected is 128 × 128 pixels, so it is resized to 28 × 28 to match the input of the InceptionResNetV2 architecture. As the dataset is very big, the recognition is conducted every 50 events. The results are analyzed as per the testing metrics described in Table 2.

**Figure 10:**
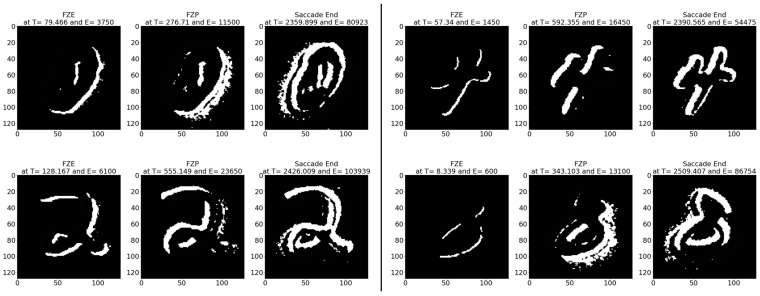
Sample MNIST-DVS test images at event (**left**) FZE, (**middle**) FPE, (**right**) saccade end.

- **Average results:** As shown in Table 6, on average the images are detected 108.69 (ms) earlier, which is around 35.51% before the full image is displayed. In terms of event sequence, the image is detected around 34.57% earlier, or 3400 events before the full image is accumulated.
  Table 7 summarizes the testing metrics per image category; only three categories are reported here for reference.
- **Sample test image results:** Figure 10 illustrates sample test images at the first zero, first perfect, and end of saccade events. It can be noticed that at the FZE images, the details of the image are not yet displayed; however, the network can still recognize the image with an average probability of 61.65%.

### 5.3. FLASH-MNIST

The same trained neural network in Section 5.2 is used to test this datatset.

The 10,000 (test dataset) raw event-based recording images are selected from the FLASH-MNIST dataset. The set contains recordings with events collected on an average time of 2103.25 (ms) and an average event count of 27,321 per recording. The size of the events collected is 128 × 128 pixels, so it is resized to 28 × 28 to match the input of the InceptionResNetV2 architecture. As the dataset is very big, the recognition is conducted every 50 events. The results are analyzed as per the testing metrics described in Table 2.

**Figure 11:**
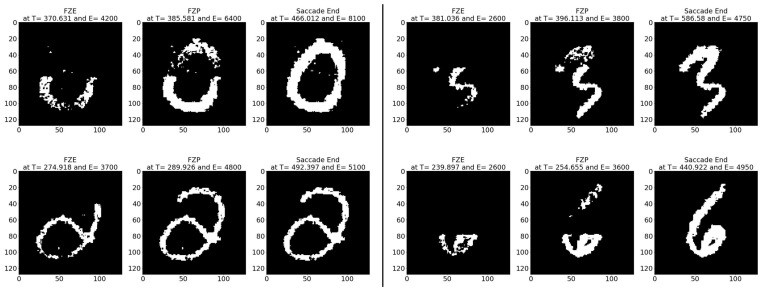
Sample FLASH-MNIST test images at event (**left**) FZE, (**middle**) FPE, (**right**) saccade end.

**Figure 12:**
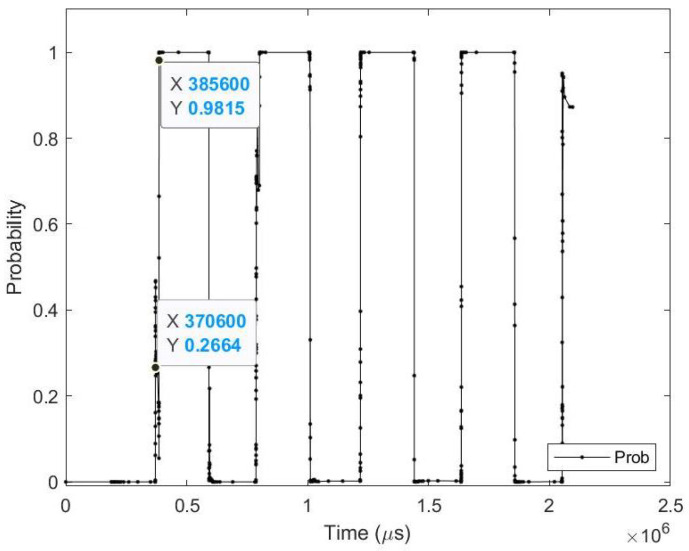
Testing result of FLASH-MNIST class 0 image using time sequence.

- **Average results:** As shown in Table 8, on average the images are detected 5.76 (ms) earlier, which is around 1.72% before the full image is displayed at the end of the saccade. In terms of event sequence, the image is detected around 8.43% earlier, or 1883 events before the end of the saccade image is accumulated. It is also noted that the average zero time and perfect time are both below 420.65 (ms), which is the duration of the first saccade.
  Table 9 summarizes the testing metrics per image category; only three categories are reported here for reference.
- **Sample test image results:** Figure 11 illustrates sample test images at the first zero, first perfect, and end of saccade events. It can be noticed that with the FZE images, the details of the image are not yet displayed; however, the network can still recognize the image with an average probability of 66.80%. The probability keeps increasing as more events are processed, as illustrated in Figure 12.
  The result of processing a single raw image file from class (0), which is shown in Figure 11 (first row), is discussed here in detail. The selected raw file contains an event count of 38,081 events which are collected on a duration of 2095.34 (ms). Each saccade is 419.07 (ms). As the events are processed, the image is updated and feedforwarded to the InceptionResNetV2 trained network. The network predicts the category of the image and provides probability against the 10 classes. Figure 12 illustrates the probability of the image (black line) against the time sequence. As discussed above, as more events are processed, the probability increases. For this image, the network is able to detect the zero and perfect event as described in Table 10.

### 5.4. N-MNIST

The same trained neural network in Section 5.2 is used to test this datatset. The 10,000 (test dataset) raw event-based recording images are selected from the N-MNIST dataset. The set contains recordings with events collected on an average time of 306.20 (ms) and an average event count of 4204 per recording. The size of the events collected is 34 × 34 pixels, so it is resized to 28 × 28 to match the input of the InceptionResNetV2 architecture. As the dataset is smaller than previous ones, the recognition is conducted every 10 events. The results are analyzed as per the testing metrics described in Table 2.

**Figure 13:**
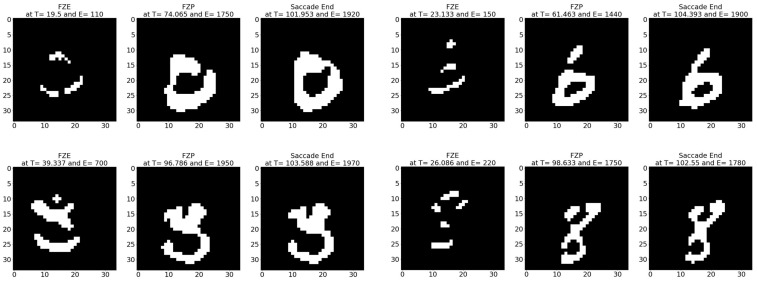
Sample N-MNIST test images at event (**left**) FZE, (**middle**) FPE, (**right**) saccade end.

**Figure 14:**
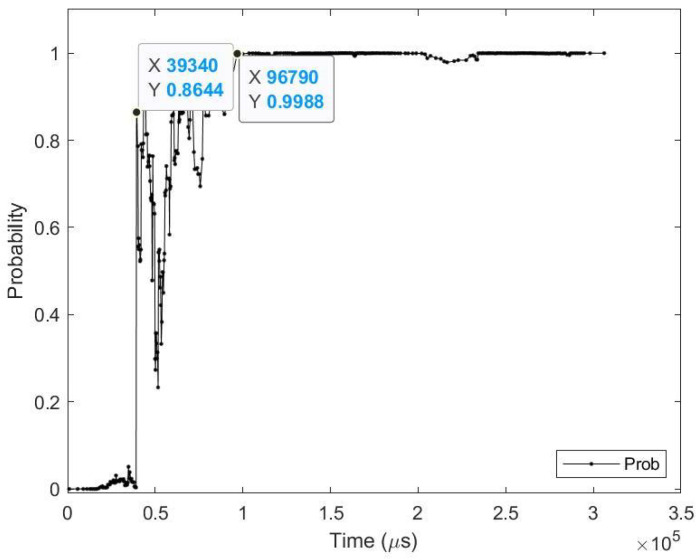
Testing result of N-MNIST class 3 image using time sequence.

- **Average results:** As shown in Table 11, on average the images are detected 7.89 (ms) earlier, which is around 19.91% before the full image is captured perfectly in FPE. In terms of event sequence, the image is detected around 32.26% earlier, or 167 events before the end of the image is accumulated in FPE. It is also noted that the average zero time and perfect time are both below 102.07 (ms), which is the duration of the first saccade.
  Table 12 summarizes the testing metrics per image category; only three categories are reported here for reference.
- **Sample test image results:** Figure 13 illustrates sample test images at the first zero, first perfect, and end of saccade events. It can be noticed that with the FZE images, the details of the image are not yet displayed; however, the network can still recognize the image with an average probability of 67.09%. The probability keeps increasing as more events are processed as illustrated in Figure 14.
  The result of processing a single raw image file from class (3), which is shown in Figure 13 (second row), is discussed here in detail. The selected raw file contains an event count of 5857 events which are collected at a duration of 306.17 (ms). Each saccade is 102.07 (ms). As the events are processed, the image is updated and feedforwarded to the InceptionResNetV2 trained network. The network predicts the category of the image and provides probability against the 10 classes. Figure 14 illustrates the probability of the image (black line) against the time sequence. As discussed above, as more events are processed, the probability increases. For this image, the network is able to detect the zero and perfect event as described in Table 13.

### 5.5. CIFAR-10

As stated in Section 4, the InceptionResNetV2 is trained on the original CIFAR-10 dataset. To calculate its accuracy, the trained model is tested on 10,000 of the original CIFAR-10 dataset and reports an accuracy of 90.18%.

The 5509 (test dataset) raw event-based recording images are selected from the CIFAR-10 DVS dataset. The set contains recordings with events collected on an average time of 1300 (ms) and an average event count of 189,145 per recording. The size of the events collected is 128 × 128 pixels, so it is resized to 32 × 32 to match the input of the InceptionResNetV2 architecture. As the dataset is very large, the recognition is conducted every 100 events. The results are analyzed as per the testing metrics described in Table 2.

**Figure 15:**
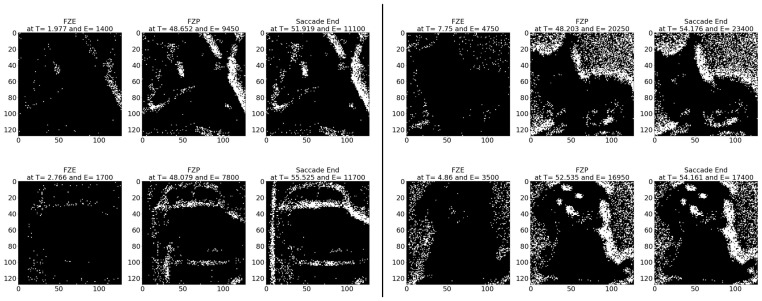
Sample CIFAR-10 DVS test images at event (**left**) FZE, (**middle**) FPE, (**right**) saccade end. Row (1) left: airplane, right: cat. Row (2) left: automobile, right: dog.

**Figure 16:**
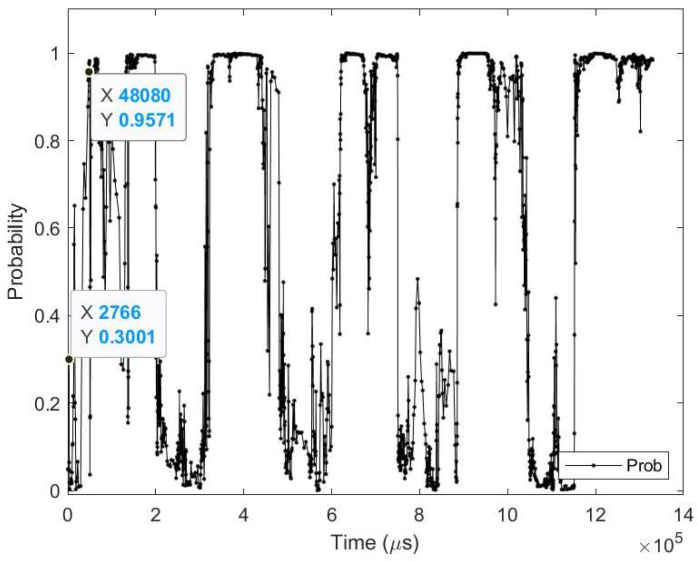
Testing result of CIFAR-10 DVS class (automobile) image using time sequence.

- **Average results:** As shown in Table 14, on average the images are detected 82.12 (ms) earlier, which is around 66.54% before the full image is captured perfectly in FPE. In terms of event sequence, the image is detected around 60.57% earlier, or 14,239 events before the end of the image is accumulated in FPE.
  Table 15 summarizes the testing metrics per image category; only three categories are reported here for reference.
- **Sample test image results:** Figure 15 illustrates sample test images at the first zero, first perfect, and end of saccade events. It can be noticed that at the FZE images, the details of the image are not yet displayed; however, the network can still recognize the image with an average probability of 45.0%. The probability keeps increasing as more events are processed as illustrated in Figure 16.
  The result of processing a single raw image file from class (automobile), which is shown in Figure 15 (second row), is discussed here in detail. The selected raw file contains an event count of 230,283 events which are collected on a duration of 1330 (ms). Each saccade is 55.53 (ms). As the events are processed, the image is updated and feedforwarded to the InceptionResNetV2 trained network. The network predicts the category of the image and provides probability against the 10 classes. Figure 16 illustrates the probability of the image (black line) against the time sequence. As discussed above, as more events are processed, the probability increases. For this image, the network is able to detect the zero and perfect events as described in Table 16. It is also noted that the zero time and perfect time are both below 55.53 (ms), which is the duration of the first saccade.

### 5.6. N-Caltech 101

As stated in Section 4, the ResNet50 is trained on the original Caltech-101 dataset. To calculate its accuracy, the trained model is tested on 20% of the original Caltech101 dataset and reports an accuracy of 94.87%.

The 1601 raw event-based recording images are selected from the N-CALTECH101 dataset. The set contains recordings with events collected at an average time of 300.14 (ms) and an average event count of 115,298 per recording. The size of the events collected is 240 × 180 pixels, so it is resized to 224 × 224 to match the input of the ResNet50 architecture. As the dataset is very large, the recognition is conducted every 50 events. The results are analyzed as per the testing metrics described in Table 2.

**Figure 17:**
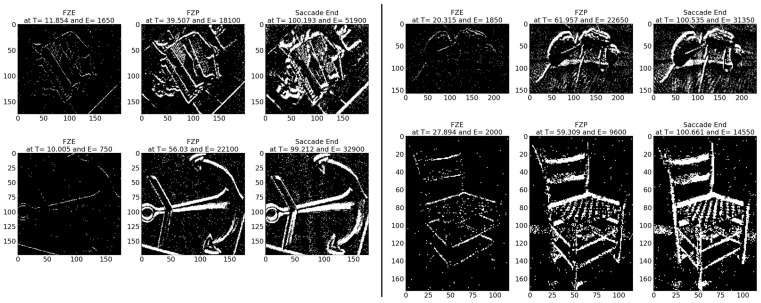
Sample N-CALTECH101 test images at event (**left**) FZE, (**middle**) FPE, (**right**) saccade end. Row (1) left: accordion, right: ant. Row (2) left: anchor, right: chair.

**Figure 18:**
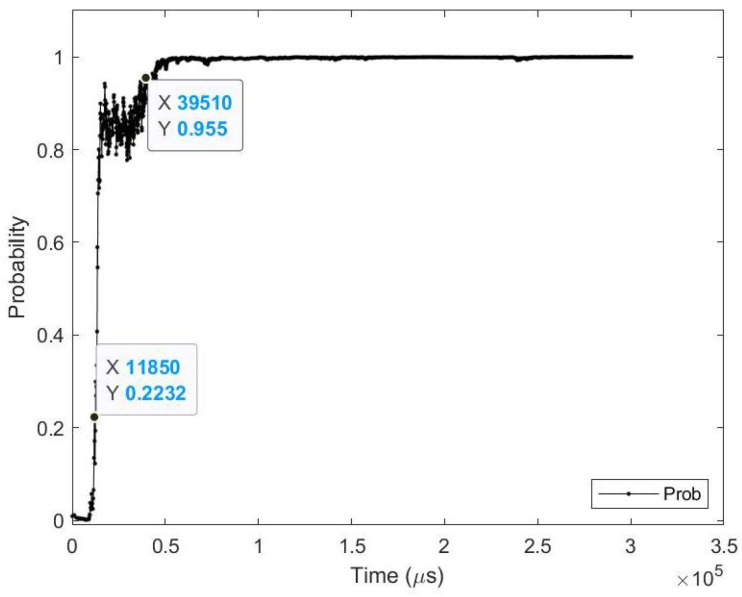
Testing result of N-CALTECH101 class (accordion) image using time sequence.

- **Average results:** As shown in Table 17, on average the images are detected 12.58 (ms) earlier, which is around 52.15% before the full image is captured perfectly in FPE. In terms of event sequence, the image is detected around 69.20% earlier, or 6074 events before the end of the image is accumulated in FPE.
  Table 18 summarizes the testing metrics per image category; only three categories are reported here for reference.
- **Sample test image results:** Figure 17 illustrates sample test images at the first zero, first perfect, and end of saccade events. It can be noticed that in the FZE images, the details of the image are not yet displayed; however, the network can still recognize the image with an average probability of 20.46%. The probability keeps increasing as more events are processed as illustrated in Figure 18.

The result of processing a single raw image file from class (automobile), which is shown in Figure 17 (first row), is discussed here in detail. The selected raw file contains an event count of 171,982 events which are collected at a duration of 300.33 (ms). Each saccade is 100.11 (ms). As the events are processed, the image is updated and feedforwarded to the ResNet50 trained network. The network predicts the category of the image and provides probability against the 101 classes. Figure 18 illustrates the probability of the image (black line) against the time sequence. As discussed above, as more events are processed the probability increases. For this image, the network is able to detect the zero and perfect event, as described in Table 19. It is also noted that the zero time and perfect time are both below 100.11 (ms) which is the duration of the first saccade.

## 6. Enhanced Early Recognition Analysis and Results

**Figure 19:**
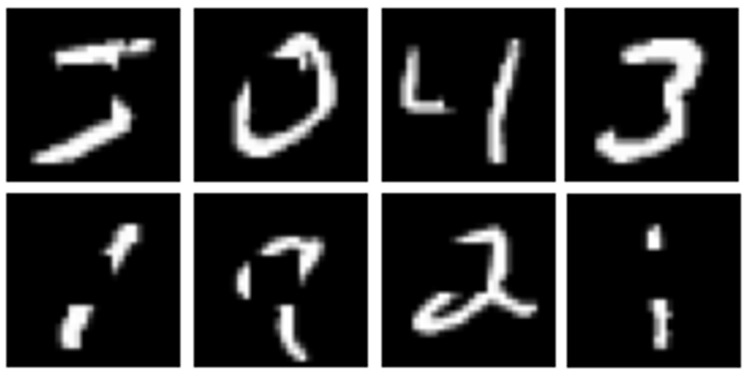
Sample of noised original MNIST dataset (partial pictures).

The recognition is enhanced by training the InceptionResNetV2 neural network in Section 4 on noisy images, as shown in Figure 19, referred to in this work as partial pictures (PP). To calculate its accuracy, the trained model is tested on 10,000 images of the noised MNIST dataset and reports an accuracy of 98.4%.

The same setup and input preprocessing techniques mentioned in previous sections are used and results are reported in this section.

### 6.1. CeleX-MNIST

- **Average results:** As shown in Table 20, on average the images are detected 13.85 (ms) earlier, which is around 47.35% before the full image is displayed. In terms of event sequence, the image is detected around 35.87% before, or 46,368 events before the full image is accumulated.
  Table 21 summarizes the testing metrics per image category; only three categories are reported here for reference.

### 6.2. MNIST-DVS

- **Average results:** As shown in Table 22, on average the images are detected 24.47 (ms) earlier, which is around 50.46% before the full image is displayed. In terms of event sequence, the image is detected around 51.42% before, or 865 events before the full image is accumulated.
  Table 23 summarizes the testing metrics per image category; only three categories are reported here for reference.

### 6.3. FLASH-MNIST

- **Average results:** As shown in Table 24, on average the images are detected 24.47 (ms) earlier, which is around 50.46% before the full image is displayed. In terms of event sequence, the image is detected around 51.42% before, or 865 events before the full image is accumulated.
  Table 25 summarizes the testing metrics per image category; only three categories are reported here for reference.

### 6.4. N-MNIST

- **Average results:** As shown in Table 26, on average the images are detected 10.79 (ms) earlier, which is around 22.49% before the full image is displayed. In terms of event sequence, the image is detected around 36.33% before, or 218 events before the full image is accumulated.
  Table 27 summarizes the testing metrics per image category; only three categories are reported here for reference.

## 7. Conclusions

In this work, fast and early object recognition for real-time applications is explored by using event-based imagers instead of full-frame cameras. The main concept is to be able to recognize an image before it is fully displayed, using events sequence rather than full-frame images. This technique allows us to decrease the time and processing power required for object recognition, leading to lower power consumption as well.

First, the InceptionResNetV2 and RestNet50 neural networks are trained on the original MNIST, CIFAR-10, and Caltech101 datasets and then tested using a pre-collected CeleX-MNIST, MNIST-DVS, FLASH-MNIST, N-MNIST, DIFAR-10, and N-Caltech101 datasets using an event-based imager. The testing metrics are based on calculating how early the network can detect an image before the full-frame image is captured.

As summarized in Table 28, we notice that on average for all the datasets, we were able to recognize an image 38.7 (ms) earlier, which is a reduction of 34% of the time needed. Less processing is also required for the image recognized 9460 events earlier, which is 37% less.These early timings are compared to the first perfect event, which is when the algorithm can detect an image with an accuracy of 95%. However, this is not when the last event is displayed. The last is event is received at the end of the saccade. The time difference between the first zero and saccade end on average is 603 (ms), excluding CIFAR-10, which did not perform well. In other words, we are able to detect an image 69.1% earlier.

Furthermore, the same neural network architecture is then trained on partial pictures to explore enhancing the early recognition time (FZE) and the saccade difference time, which refers to when the last event is received. This test was only performed on MNIST datasets, and as shown in Table 29, it can be noticed that FZE is reduced and is detected at 104.2 (ms) instead of 160.4 (ms). Moreover, the time difference between the first zero and saccade end is also reduced, and on average is 789.1 (ms); in other words, we are able to detect an image 71.0% earlier.

## Figures and Tables

**Table 1:** Dataset statistics results.

Dataset	Average					Min X	Min Y	Max X	Max Y
	Total E	Events	ON E	OFF E	Saccade T				
**CeleX-MNIST [this work]**	631	420,546	268,129	152,417	631	0	1279	0	799
**MNIST-DVS (Scale 4)** [19]	2261	17,011	8662	8349	2261.11	0	127	0	127
**MNIST-DVS (Scale 8)** [19]	2371	43,764	21,841	21,922	2370.70	0	127	0	127
**MNIST-DVS (Scale 16)** [19]	2412	103,144	50,985	52,158	2411.81	0	127	0	127
**FLASH-MNIST (Test)** [19]	2103	27,321	16,410	10,910	420.65	1	128	1	128
**FLASH-MNIST (Train)** [19]	2147	26,713	16,018	10,694	429.35	1	128	1	128
**N-MNIST (Test)** [20]	306	4204	2116	2087	102.07	1	34	1	34
**N-MNIST (Train)** [20]	307	4172	2088	2084	102.17	1	34	1	34
**CIFAR-10** [21]	1300	183,145	76,870	106,276	54.19	0	127	0	127
**N-Caltech 101** [20]	300	115,298	58,289	57,009	100.05	1	233	1	173

**Table 2:** Testing metrics.

**Zero_Time**	Average time of FZE (ms).
**Zero_Event**	Average event of FZE.
**Zero_Prob**	Average probability of FZE.
**Perfect_Time**	Average time of FPE (ms).
**Perfect_Event**	Average event of FPE.
**Perfect_Prob**	Average probability of FPE.
**Time_Diff**	Average time difference between FPE and FZE, which determines how early an image is detected in terms of time (ms).
**Time %**	Average time difference percentage between FPE and FZE, which determines how early an image is detected in terms of time.
**Event_Diff**	Average event difference between FPE and FZE, which determines how early an image is detected in terms of event number.
**Event %**	Average event difference percentage between FPE and FZE, which determines how early an image is detected in terms of event number
**Sacc_Time_Diff**	Average time difference between saccade end and FZE, which determines how early an image is detected in terms of time (ms).

**Table 3:** Average results for CeleX-MNIST test images.

**Zero_Time**	39.69 (ms)	**Zero_Prob**	56.20%
**Perfect_Time**	51.46 (ms)	**Perfect_Prob**	78.72%
**Zero_Event**	153,598 (events)	**Perfect_Event**	172,121 (events)
**Time_Diff**	14.81 (ms)	**Time %**	28.78%
**Event_Diff**	32,558 (events)	**Event %**	18.92%
**Sacc_Diff**	613.73 (ms)	**Sacc %**	93.98%

**Table 4:** Average results for (3) Categories of CeleX-MNIST test images.

**Class 3 (30 images)**			
**Zero_Time**	51.81 (ms)	**Zero_Prob**	47.72%
**Perfect_Time**	64.18 (ms)	**Perfect_Prob**	74.17%
**Zero_Event**	198,633 (events)	**Perfect_Event**	222,683 (events)
**Time_Diff**	17.00 (ms)	**Time %**	26.48%
**Event_Diff**	36,016 (events)	**Event %**	16.17%
**Sacc_Diff**	621.71 (ms)	**Sacc %**	92.31%
**Class 6 (30 images)**			
**Zero_Time**	65.37 (ms)	**Zero_Prob**	50.95%
**Perfect_Time**	80.19 (ms)	**Perfect_Prob**	73.17%
**Zero_Event**	189,450 (events)	**Perfect_Event**	213,350 (events)
**Time_Diff**	14.97 (ms)	**Time %**	18.66%
**Event_Diff**	29,550 (events)	**Event %**	13.85%
**Sacc_Diff**	636.20 (ms)	**Sacc %**	90.68%
**Class 9 (30 images)**			
**Zero_Time**	38.11 (ms)	**Zero_Prob**	49.71%
**Perfect_Time**	54.68 (ms)	**Perfect_Prob**	80.85%
**Zero_Event**	158,433 (events)	**Perfect_Event**	185,833 (events)
**Time_Diff**	20.46 (ms)	**Time %**	37.42%
**Event_Diff**	40,066 (events)	**Event %**	21.56%
**Sacc_Diff**	557.90 (ms)	**Sacc %**	93.61%

**Table 5:** Average results for CeleX-MNIST class 2 test image.

**Zero_Time**	3.80 (ms)	**Zero_Prob**	55.77%
**Perfect_Time**	101.57 (ms)	**Perfect_Prob**	96.65%
**Zero_Event**	221,000 (events)	**Perfect_Event**	505,000 (events)
**Time_Diff**	97.7 (ms)	**Time %**	96.26%
**Event_Diff**	284,000 (events)	**Event %**	56.24%

**Table 6:** Average results for MNIST-DVS test images.

**Zero_Time**	235.45 (ms)	**Zero_Prob**	61.65%
**Perfect_Time**	306.11 (ms)	**Perfect_Prob**	87.22%
**Zero_Event**	7616 (events)	**Perfect_Event**	9834 (events)
**Time_Diff**	108.69 (ms)	**Time %**	35.51%
**Event_Diff**	3400 (events)	**Event %**	34.57%
**Sacc_Diff**	2176.37 (ms)	**Sacc %**	90.24%

**Table 7:** Average results for (3) categories of MNIST-DVS test images.

**Class 2 (1000 images)**			
**Zero_Time**	546.72 (ms)	**Zero_Prob**	47.71%
**Perfect_Time**	578.64 (ms)	**Perfect_Prob**	82.70%
**Zero_Event**	17,582 (events)	**Perfect_Event**	18,303 (events)
**Time_Diff**	101.3 (ms)	**Time %**	17.51%
**Event_Diff**	1865 (events)	**Event %**	16.31%
**Sacc_Diff**	1865.09 (ms)	**Sacc %**	77.33%
**Class 4 (1000 images)**			
**Zero_Time**	188.48 (ms)	**Zero_Prob**	65.67%
**Perfect_Time**	322.13 (ms)	**Perfect_Prob**	78.24%
**Zero_Event**	5185 (events)	**Perfect_Event**	8546 (events)
**Time_Diff**	179.34 (ms)	**Time %**	55.67%
**Event_Diff**	4633 (events)	**Event %**	54.21%
**Sacc_Diff**	2223.33 (ms)	**Sacc %**	92.19%
**Class 6 (1000 images)**			
**Zero_Time**	452.23 (ms)	**Zero_Prob**	50.70%
**Perfect_Time**	515.07 (ms)	**Perfect_Prob**	79.66%
**Zero_Event**	12,301 (events)	**Perfect_Event**	13,844 (events)
**Time_Diff**	146.05 (ms)	**Time %**	28.35%
**Event_Diff**	3650 (events)	**Event %**	26.36%
**Sacc_Diff**	1959.59 (ms)	**Sacc %**	81.25%

**Table 8:** Average results for FLASH-MNIST test images.

**Zero_Time**	336.79 (ms)	**Zero_Prob**	66.80%
**Perfect_Time**	335.82 (ms)	**Perfect_Prob**	96.00%
**Zero_Event**	3581 (events)	**Perfect_Event**	3842 (events)
**Time_Diff**	5.76 (ms)	**Time %**	1.72%
**Event_Diff**	324 (events)	**Event %**	8.43%
**Sacc_Diff**	83.86 (ms)	**Sacc %**	19.94%

**Table 9:** Average results for (3) categories of FLASH-MNIST test images.

**Class 0 (980 images)**			
**Zero_Time**	372.41 (ms)	**Zero_Prob**	63.15%
**Perfect_Time**	375.58 (ms)	**Perfect_Prob**	97.64%
**Zero_Event**	4402 (events)	**Perfect_Event**	4860 (events)
**Time_Diff**	3.17 (ms)	**Time %**	0.84%
**Event_Diff**	458 (events)	**Event %**	9.41%
**Sacc_Diff**	51.60 (ms)	**Sacc %**	12.20%
**Class 3 (1010 images)**			
**Zero_Time**	374.31 (ms)	**Zero_Prob**	60.56%
**Perfect_Time**	372.17 (ms)	**Perfect_Prob**	96.87%
**Zero_Event**	4229 (events)	**Perfect_Event**	4532 (events)
**Time_Diff**	2.36 (ms)	**Time %**	0.63%
**Event_Diff**	329 (events)	**Event %**	7.27%
**Sacc_Diff**	52.80 (ms)	**Sacc %**	12.60%
**Class 7 (1028 images)**			
**Zero_Time**	369.08 (ms)	**Zero_Prob**	65.89%
**Perfect_Time**	366.89 (ms)	**Perfect_Prob**	93.78%
**Zero_Event**	3030 (events)	**Perfect_Event**	3313 (events)
**Time_Diff**	7.29 (ms)	**Time %**	1.99%
**Event_Diff**	350 (events)	**Event %**	10.57%
**Sacc_Diff**	42.62 (ms)	**Sacc %**	10.14%

**Table 10:** Average results for FLASH-MNIST class 0 test image.

**Zero_Time**	370.63 (ms)	**Zero_Prob**	26.64%
**Perfect_Time**	385.58 (ms)	**Perfect_Prob**	98.15%
**Zero_Event**	4200 (events)	**Perfect_Event**	6400 (events)
**Time_Diff**	14.95 (ms)	**Time %**	3.88%
**Event_Diff**	2200 (events)	**Event %**	34.38%

**Table 11:** Average results for N-MNIST test images.

**Zero_Time**	32.47 (ms)	**Zero_Prob**	67.09%
**Perfect_Time**	39.65 (ms)	**Perfect_Prob**	96.40%
**Zero_Event**	358 (events)	**Perfect_Event**	516 (events)
**Time_Diff**	7.89 (ms)	**Time %**	19.91%
**Event_Diff**	167 (events)	**Event %**	32.26%
**Sacc_Diff**	69.60 (ms)	**Sacc %**	68.18%

**Table 12:** Average results for (3) categories of N-MNIST test images.

**Class 5 (892 images)**			
**Zero_Time**	25.17 (ms)	**Zero_Prob**	66.09%
**Perfect_Time**	29.88 (ms)	**Perfect_Prob**	97.31%
**Zero_Event**	207 (events)	**Perfect_Event**	313 (events)
**Time_Diff**	4.74 (ms)	**Time %**	15.86%
**Event_Diff**	107 (events)	**Event %**	34.07%
**Sacc_Diff**	76.90 (ms)	**Sacc %**	75.34%
**Class 8 (974 images)**			
**Zero_Time**	34.89 (ms)	**Zero_Prob**	67.58%
**Perfect_Time**	44.24 (ms)	**Perfect_Prob**	96.78%
**Zero_Event**	396 (events)	**Perfect_Event**	598 (events)
**Time_Diff**	9.96 (ms)	**Time %**	22.51%
**Event_Diff**	210 (events)	**Event %**	35.11%
**Sacc_Diff**	67.18 (ms)	**Sacc %**	65.81%
**Class 9 (1009 images)**			
**Zero_Time**	31.75 (ms)	**Zero_Prob**	60.02%
**Perfect_Time**	36.82 (ms)	**Perfect_Prob**	95.01%
**Zero_Event**	252 (events)	**Perfect_Event**	357 (events)
**Time_Diff**	6.33 (ms)	**Time %**	17.19%
**Event_Diff**	116 (events)	**Event %**	32.36%
**Sacc_Diff**	70.32 (ms)	**Sacc %**	68.90%

**Table 13:** Average results for N-MNIST class 3 test image.

**Zero_Time**	39.34 (ms)	**Zero_Prob**	86.44%
**Perfect_Time**	96.79 (ms)	**Perfect_Prob**	99.88%
**Zero_Event**	700 (events)	**Perfect_Event**	1950 (events)
**Time_Diff**	57.45 (ms)	**Time %**	59.36%
**Event_Diff**	1250 (events)	**Event %**	64.10%

**Table 14:** Average results for CIFAR-10 DVS test images.

**Zero_Time**	88.96 (ms)	**Zero_Prob**	44.99%
**Perfect_Time**	123.42 (ms)	**Perfect_Prob**	58.45%
**Zero_Event**	18,519 (events)	**Perfect_Event**	23,507 (events)
**Time_Diff**	82.12 (ms)	**Time %**	66.54%
**Event_Diff**	14,239 (events)	**Event %**	60.57%

**Table 15:** Average results for (3) categories of CIFAR-10 DVS test images.

**Class Airplane (1000 images)**			
**Zero_Time**	11.88 (ms)	**Zero_Prob**	54.40%
**Perfect_Time**	43.97 (ms)	**Perfect_Prob**	92.69%
**Zero_Event**	3960 (events)	**Perfect_Event**	9679 (events)
**Time_Diff**	33.77 (ms)	**Time %**	72.98%
**Event_Diff**	6062 (events)	**Event %**	59.09%
**Class Bird (380 images)**			
**Zero_Time**	129.43 (ms)	**Zero_Prob**	43.95%
**Perfect_Time**	155.67 (ms)	**Perfect_Prob**	45.78%
**Zero_Event**	26,341 (events)	**Perfect_Event**	26,521 (events)
**Time_Diff**	32.09 (ms)	**Time %**	16.86%
**Event_Diff**	180 (events)	**Event %**	0.68%
**Class Cat (1000 images)**			
**Zero_Time**	93.39 (ms)	**Zero_Prob**	52.39%
**Perfect_Time**	180.91 (ms)	**Perfect_Prob**	65.54%
**Zero_Event**	22,720 (events)	**Perfect_Event**	35,133 (events)
**Time_Diff**	87.52 (ms)	**Time %**	48.38%
**Event_Diff**	12,414 (events)	**Event %**	35.33%

**Table 16:** Average results for CIFAR-10 DVS class (automobile) test image.

**Zero_Time**	2.77 (ms)	**Zero_Prob**	30.01%
**Perfect_Time**	48.08 (ms)	**Perfect_Prob**	95.71%
**Zero_Event**	1700 (events)	**Perfect_Event**	7800 (events)
**Time_Diff**	45.31 (ms)	**Time %**	94.25%
**Event_Diff**	6100 (events)	**Event %**	78.21%

**Table 17:** Average results for N-CALTECH101 test images.

**Zero_Time**	26.60 (ms)	**Zero_Prob**	20.46%
**Perfect_Time**	24.11 (ms)	**Perfect_Prob**	45.46%
**Zero_Event**	7701 (events)	**Perfect_Event**	8778 (events)
**Time_Diff**	12.58 (ms)	**Time %**	52.15%
**Event_Diff**	6074 (events)	**Event %**	69.20%
**Sacc_Diff**	73.45 (ms)	**Sacc %**	73.41%

**Table 18:** Average results for (3) categories of N-CALTECH101 test images.

**Class menorah (87 images)**			
**Zero_Time**	18.17 (ms)	**Zero_Prob**	18.33%
**Perfect_Time**	427.92 (ms)	**Perfect_Prob**	92.45%
**Zero_Event**	3836 (events)	**Perfect_Event**	8069.54 (events)
**Time_Diff**	11.24 (ms)	**Time %**	40.25%
**Event_Diff**	5252 (events)	**Event %**	65.09%
**Sacc_Diff**	81.88 (ms)	**Sacc %**	81.84%
**Class stop_sign (64 images)**			
**Zero_Time**	45.42 (ms)	**Zero_Prob**	22.70%
**Perfect_Time**	50.11 (ms)	**Perfect_Prob**	52.7%
**Zero_Event**	15,113 (events)	**Perfect_Event**	19,893 (events)
**Time_Diff**	27.71 (ms)	**Time %**	55.31%
**Event_Diff**	11,881 (events)	**Event %**	59.73%
**Sacc_Diff**	54.63 (ms)	**Sacc %**	54.60%
**Class yin_yang (60 images)**			
**Zero_Time**	15.96 (ms)	**Zero_Prob**	21.60%
**Perfect_Time**	38.39 (ms)	**Perfect_Prob**	70.39%
**Zero_Event**	3200 (events)	**Perfect_Event**	9685 (events)
**Time_Diff**	28.32 (ms)	**Time %**	73.76%
**Event_Diff**	8649 (events)	**Event %**	89.30%
**Sacc_Diff**	84.09 (ms)	**Sacc %**	84.05%

**Table 19:** Average results for N-CALTECH101 class (accordion) test image.

**Zero_Time**	11.85 (ms)	**Zero_Prob**	22.32%
**Perfect_Time**	39.51 (ms)	**Perfect_Prob**	95.50%
**Zero_Event**	1650 (events)	**Perfect_Event**	18,100 (events)
**Time_Diff**	27.65 (ms)	**Time %**	70.00%
**Event_Diff**	16,450 (events)	**Event %**	90.88%

**Table 20:** Average PP results for CeleX-MNIST test images.

**Zero_Time**	18.17 (ms)	**Zero_Prob**	58.45%
**Perfect_Time**	28.64 (ms)	**Perfect_Prob**	81.29%
**Zero_Event**	104,198 (events)	**Perfect_Event**	133,067 (events)
**Time_Diff**	13.85 (ms)	**Time %**	47.35%
**Event_Diff**	46,368 (events)	**Event %**	35.87%
**Sacc_Diff**	635.24 (ms)	**Sacc %**	97.23%

**Table 21:** Average PP results for (3) categories of CeleX-MNIST test images.

**Class 3 (30 images)**			
**Zero_Time**	21.21 (ms)	**Zero_Prob**	56.17%
**Perfect_Time**	25.50 (ms)	**Perfect_Prob**	81.07%
**Zero_Event**	127,317 (events)	**Perfect_Event**	141,867 (events)
**Time_Diff**	8.75 (ms)	**Time %**	34.31%
**Event_Diff**	38,983 (events)	**Event %**	27.48%
**Sacc_Diff**	652.31 (ms)	**Sacc %**	96.85%
**Class 6 (30 images)**			
**Zero_Time**	25.95 (ms)	**Zero_Prob**	49.57%
**Perfect_Time**	44.33 (ms)	**Perfect_Prob**	85.98%
**Zero_Event**	106,300 (events)	**Perfect_Event**	164,700 (events)
**Time_Diff**	21.04 (ms)	**Time %**	47.47%
**Event_Diff**	74,933 (events)	**Event %**	45.50%
**Sacc_Diff**	675.63 (ms)	**Sacc %**	96.30%
**Class 9 (30 images)**			
**Zero_Time**	4.89 (ms)	**Zero_Prob**	66.06%
**Perfect_Time**	20.62 (ms)	**Perfect_Prob**	95.64%
**Zero_Event**	34,817 (events)	**Perfect_Event**	103,483 (events)
**Time_Diff**	15.73 (ms)	**Time %**	76.28%
**Event_Diff**	68,717 (events)	**Event %**	66.40%
**Sacc_Diff**	591.12 (ms)	**Sacc %**	99.18%

**Table 22:** Average PP results for MNIST-DVS test images.

**Zero_Time**	29.85 (ms)	**Zero_Prob**	62.87%
**Perfect_Time**	43.81 (ms)	**Perfect_Prob**	79.49%
**Zero_Event**	998 (events)	**Perfect_Event**	1495 (events)
**Time_Diff**	24.47 (ms)	**Time %**	50.46%
**Event_Diff**	865 (events)	**Event %**	51.42%
**Sacc_Diff**	2366.45 (ms)	**Sacc %**	98.76%

**Table 23:** Average PP results for (3) categories of MNIST-DVS test images.

**Class 2 (1000 images)**			
**Zero_Time**	76.47 (ms)	**Zero_Prob**	44.14%
**Perfect_Time**	91.50 (ms)	**Perfect_Prob**	61.66%
**Zero_Event**	2308 (events)	**Perfect_Event**	2696 (events)
**Time_Diff**	48.53 (ms)	**Time %**	53.04%
**Event_Diff**	1455 (events)	**Event %**	53.99%
**Sacc_Diff**	2313.96 (ms)	**Sacc %**	96.80%
**Class 4 (1000 images)**			
**Zero_Time**	23.18 (ms)	**Zero_Prob**	72.23%
**Perfect_Time**	36.18 (ms)	**Perfect_Prob**	91.88%
**Zero_Event**	610 (events)	**Perfect_Event**	961 (events)
**Time_Diff**	16.44 (ms)	**Time %**	45.43%
**Event_Diff**	438 (events)	**Event %**	45.63%
**Sacc_Diff**	2364.61 (ms)	**Sacc %**	99.03%
**Class 6 (1000 images)**			
**Zero_Time**	21.75 (ms)	**Zero_Prob**	59.56%
**Perfect_Time**	34.78 (ms)	**Perfect_Prob**	75.12%
**Zero_Event**	547 (events)	**Perfect_Event**	896 (events)
**Time_Diff**	20.09 (ms)	**Time %**	57.76%
**Event_Diff**	527 (events)	**Event %**	58.85%
**Sacc_Diff**	2364.90 (ms)	**Sacc %**	99.09%

**Table 24:** Average PP results for FLASH-MNIST test images.

**Zero_Time**	335.37 (ms)	**Zero_Prob**	72.52%
**Perfect_Time**	338.37 (ms)	**Perfect_Prob**	97.33%
**Zero_Event**	3460 (events)	**Perfect_Event**	3820 (events)
**Time_Diff**	5.73 (ms)	**Time %**	1.51%
**Event_Diff**	391 (events)	**Event %**	9.33%
**Sacc_Diff**	83.86 (ms)	**Sacc %**	19.94%

**Table 25:** Average PP results for (3) categories of FLASH-MNIST test images.

**Class 0 (980 images)**			
**Zero_Time**	371.17 (ms)	**Zero_Prob**	61.54%
**Perfect_Time**	374.80 (ms)	**Perfect_Prob**	97.49%
**Zero_Event**	4862 (events)	**Perfect_Event**	5516 (events)
**Time_Diff**	3.63 (ms)	**Time %**	0.97%
**Event_Diff**	654 (events)	**Event %**	11.85%
**Sacc_Diff**	51.60 (ms)	**Sacc %**	12.20%
**Class 3 (1010 images)**			
**Zero_Time**	366.30 (ms)	**Zero_Prob**	69.26%
**Perfect_Time**	371.96 (ms)	**Perfect_Prob**	97.55%
**Zero_Event**	4723 (events)	**Perfect_Event**	5059 (events)
**Time_Diff**	7.21 (ms)	**Time %**	1.94%
**Event_Diff**	370 (events)	**Event %**	7.31%
**Sacc_Diff**	52.80 (ms)	**Sacc %**	12.60%
**Class 7 (1028 images)**			
**Zero_Time**	377.81 (ms)	**Zero_Prob**	68.64%
**Perfect_Time**	384.87 (ms)	**Perfect_Prob**	95.39%
**Zero_Event**	3368 (events)	**Perfect_Event**	3991 (events)
**Time_Diff**	16.79 (ms)	**Time %**	4.36%
**Event_Diff**	718 (events)	**Event %**	17.98%
**Sacc_Diff**	42.62 (ms)	**Sacc %**	10.14%

**Table 26:** Average PP results for N-MNIST test images.

**Zero_Time**	33.33 (ms)	**Zero_Prob**	71.49%
**Perfect_Time**	43.28 (ms)	**Perfect_Prob**	96.38%
**Zero_Event**	383 (events)	**Perfect_Event**	592 (events)
**Time_Diff**	10.79 (ms)	**Time %**	22.49%
**Event_Diff**	218 (events)	**Event %**	36.33%
**Sacc_Diff**	69.11 (ms)	**Sacc %**	67.46%

**Table 27:** Average PP results for (3) categories of N-MNIST test images.

**Class 5 (892 images)**			
**Zero_Time**	35.02 (ms)	**Zero_Prob**	69.90%
**Perfect_Time**	47.41 (ms)	**Perfect_Prob**	9713%
**Zero_Event**	476 (events)	**Perfect_Event**	765 (events)
**Time_Diff**	12.92 (ms)	**Time %**	27.24%
**Event_Diff**	298 (events)	**Event %**	38.92%
**Sacc_Diff**	67.59 (ms)	**Sacc %**	65.87%
**Class 8 (974 images)**			
**Zero_Time**	49.57 (ms)	**Zero_Prob**	71.91%
**Perfect_Time**	59.69 (ms)	**Perfect_Prob**	96.82%
**Zero_Event**	854 (events)	**Perfect_Event**	1077 (events)
**Time_Diff**	11.47 (ms)	**Time %**	19.21%
**Event_Diff**	239 (events)	**Event %**	22.18%
**Sacc_Diff**	52.91 (ms)	**Sacc %**	51.63%
**Class 9 (1009 images)**			
**Zero_Time**	22.55 (ms)	**Zero_Prob**	71.26%
**Perfect_Time**	30.42 (ms)	**Perfect_Prob**	98.05%
**Zero_Event**	117 (events)	**Perfect_Event**	236 (events)
**Time_Diff**	78.65 (ms)	**Time %**	25.86%
**Event_Diff**	119 (events)	**Event %**	50.53%
**Sacc_Diff**	79.99 (ms)	**Sacc %**	78.01%

**Table 28:** Dataset recognition results summary.

	Dataset							Total Average
	MNIST					CIFAR 10	N-CALTECH 101	
	CeleX	DVS	FLASH	N	avg			
**Zero**								
Time (ms)	36.7	235.5	336.8	32.5	160.4	89.0	26.6	126.2
Prob %	56.2	61.7	66.8	67.1	63.0	45.0	20.5	52.9
Event	153,598	7616	3518	358	41,273	18,519	7701	31,885
**Perfect**								
Time (ms)	51.5	306.1	335.9	39.65	183.3	123.4	24.1	146.8
Prob %	78.7	87.2	96.0	96.4	89.6	58.5	45.5	77.1
Event	172,121	7616	3842	516	46,024	23,507	8778	36,063
**Time**								
Diff (ms)	14.8	108.7	5.8	7.9	34.3	82.1	12.6	38.7
Diff %	28.8	35.5	1.7	19.9	21.5	66.5	52.2	34.1
**Event**								
Diff	32,558	3400	324	167	9112	14,239	6074	9460
Diff %	18.9	34.6	8.43	32.3	23.6	60.6	69.2	37.3
**Saccade**								
Diff	613.7	2176.4	83.9	69.6	735.9	-	73.5	603.4
Diff %	93.9	90.2	19.9	68.2	68.1	-	73.4	69.1

**Table 29:** Dataset partial pictures recognition results summary.

	Dataset				Average
	MNIST				
	CeleX	DVS	FLASH	N	
**Zero**					
Time (ms)	18.2	29.8	335.4	33.3	104.2
Prob %	58.5	62.9	73.5	71.5	66.6
Event	104,198	998	3460	383	27,260
**Perfect**					
Time (ms)	28.6	43.8	338.4	43.3	113.5
Prob %	81.3	79.5	97.3	96.4	88.6
Event	133,067	1495	3820	592	34,743
**Time**					
Diff (ms)	13.9	24.5	5.7	10.8	13.7
Diff %	47.3	50.5	1.5	22.5	30.5
**Event**					
Diff	46,368	865	391	218	11,960
Diff %	35.9	51.4	9.3	36.3	33.2
**Saccade**					
Diff	635.2	2366.5	85.6	69.1	789.1
Diff %	97.2	98.8	20.4	67.5	71.0

## Data Availability

The data supporting the reported results were conducted by the authors and are available on request from the corresponding author.
